# Upconversion Nanoparticles with Mesoporous Silica Coatings for Doxorubicin Targeted Delivery to Melanoma Cells

**DOI:** 10.3390/molecules31010074

**Published:** 2025-12-24

**Authors:** Párástu Oskoei, Rúben Afonso, Verónica Bastos, João Nogueira, Lisa-Marie Keller, Elina Andresen, Maysoon I. Saleh, Bastian Rühle, Ute Resch-Genger, Ana L. Daniel-da-Silva, Helena Oliveira

**Affiliations:** 1 CESAM–Centre for Environmental and Marine Studies, Department of Biology, University of Aveiro, Campus Universitário de Santiago, 3810-193 Aveiro, Portugal; rubenafonso96@gmail.com (R.A.); veronicabastos@ua.pt (V.B.); 2 CICECO–Aveiro Institute of Materials, Department of Chemistry, University of Aveiro, Campus Universitário de Santiago, 3810-193 Aveiro, Portugal; jh.nogueira@ua.pt (J.N.); ana.luisa@ua.pt (A.L.D.-d.-S.); 3 Federal Institute for Material Research Testing (BAM), Unter den Eichen 87, 12205 Berlin, Germany; lisa-m.keller@gmx.de (L.-M.K.); elina-andresen@web.de (E.A.); maysoon.i.saleh@gmail.com (M.I.S.); bastian.ruehle@bam.de (B.R.); ute.resch@bam.de (U.R.-G.)

**Keywords:** upconversion nanoparticles, chemotherapy, melanoma cells, targeted delivery, folic acid, mesoporous silica shell

## Abstract

Melanoma is one of the most aggressive skin cancers and requires innovative therapeutic strategies to overcome the limitations of conventional therapies. In this work, upconversion nanoparticles coated with mesoporous silica and functionalized with folic acid (UCNP@mSiO_2_-FA) were developed as a targeted nanocarrier system for the delivery of doxorubicin (DOX). The UCNPs were synthesized via thermal decomposition, coated with mesoporous silica shells, and functionalized with folic acid (FA) to enable receptor-mediated targeting. DOX was then loaded into the mesoporous silica coating by adsorption, yielding UCNP@mSiO_2_-FA-DOX. The different UCNPs were characterized for size, composition, colloidal stability, and loading and release of DOX. This comprehensive physicochemical characterization confirmed a high DOX loading efficiency and a slightly increased drug release under acidic conditions, mimicking the tumour microenvironment. In vitro assays using four melanoma cell lines (A375, B16-F10, MNT-1, and SK-MEL-28) revealed an excellent biocompatibility of UCNP@mSiO_2_-FA and a significantly higher cytotoxicity of UCNP@mSiO_2_-FA-DOX compared to unloaded UCNPs, in a dose-dependent manner. Cell cycle analysis demonstrated G2/M phase arrest after treatment with UCNP@mSiO_2_-FA-DOX, confirming its antiproliferative effect. Overall, UCNP@mSiO_2_-FA-DOX represents a promising nanoplatform for targeted melanoma therapy, combining active tumour targeting and enhanced anticancer efficacy.

## 1. Introduction

Melanoma is a type of skin cancer that develops in melanocytes, and its incidence has been increasing since 1970 [1]. In 2018, an estimated number of 290,000 new cases and 61,000 deaths were reported worldwide [2]. When detected at an early stage, melanoma is treatable with localized surgery and has an excellent 5-year relative survival rate of over 99% [3]. However, when diagnosed at advanced or metastatic stages, the survival rate decreases drastically [3]. In recent years, biomedical applications based on nanotechnology have been increasing due to the unique optical, magnetic, electronic, or mechanical properties of nanomaterials, as well as their ability to be manipulated through internal or external stimuli. These properties enable their use in both diagnosis and therapeutic systems, including targeted drug delivery, hyperthermia, and cell labelling [4,5,6,7]. Advances in nanotechnology allow nanoparticles to act as carriers for almost any type of molecule and support their controlled delivery, release, or targeted administration to specific cells or organs [8,9]. The application of nanoparticles can improve drug bioavailability, minimize organ toxicity, and reduce possible side effects [10].

In tumour-targeted drug delivery using nanoparticles, the drug is loaded into nanoparticle carriers and transported to the target site to be released. Nanoparticle-based drug delivery offers several advantages, for example, the enhanced drug accumulation in the tumour tissue through active or passive targeting mechanisms such as the enhanced permeability and retention (EPR) effect. This approach enables the use of potentially effective anti-melanoma chemotherapeutic agents that have demonstrated significant melanoma growth inhibition in vitro, such as docetaxel, paclitaxel, and doxorubicin (DOX), but their clinical application in melanoma treatment is largely hampered due to their poor solubility or stability, or their high toxicity to healthy cells [11]. When combined with triggered release strategies, this approach ideally allows non-target tissues to remain unaffected, while preserving the drug’s therapeutic benefits, thus decreasing the toxicity, side-effects, and overall impact on the patient’s life quality [12]. To enable optical monitoring of such nanoparticle drug carriers, luminescent nanocomposites can be designed. A promising approach involves the coating of a luminescent nanoparticle with a mesoporous silica shell that can be subsequently loaded with a drug [13]. Interesting candidates are lanthanide-based upconversion nanoparticles (UCNPs) that consist of an inorganic host crystal doped with luminescent lanthanide ions such as NaYF_4_:Yb,Er. These chemically inert and photostable nanomaterials exhibit a nonlinear multi-colour luminescence with characteristic emission bands in the ultraviolet (UV), visible (vis), and near-infrared (NIR) regions, resulting from sequential absorption of two or more lower energy photons. They also display a linear, downshifted emission in the short-wave infrared (SWIR) [14]. UCNPs have a high biocompatibility [15,16] and their use in biomedical research is increasing. Such systems can be exploited for the design of stimuli-responsive nanocarriers for light-activated DOX release [17] or pH-dependent drug release [18,19]. These properties make UCNPs attractive tools for pharmaceutical drug screening and bioimaging applications [20,21,22,23].

To increase the interaction between nanoparticles and cancer cells, drug nanocarriers are often functionalized with surface ligands that specifically bind to cancer-specific biomarkers such as certain proteins, surface receptors, or cellular structures overexpressed on the surface of cancer cells, thereby acting as targeting agents [24]. Folic acid (FA) has emerged as a promising targeting ligand in nanoparticle-based drug delivery systems [25]. Nearly all cells express a folate receptor on the surface; however, cancer cells tend to overexpress these receptors due to their higher needs for energy for increased cell growth and division [25], for example, as observed in SK-MEL-28 melanoma cell line [26]. Consequently, nanoparticles conjugated with FA show facilitated, receptor-mediated endocytosis, increasing the uptake by cancer cells compared to healthy cells, which in turn enhances the therapeutic efficacy of the nanoformulations while reducing their systemic toxicity [27]. Furthermore, the small size, stability, and ease of chemical modification of FA make it an ideal candidate for the surface functionalization of various nanoparticle platforms, including liposomes, polymer nanoparticles, and gold nanoparticles [25]. Some examples of the improvement of uptake by nanoplatforms that were functionalised with FA when compared to the same nanoplatforms without this functionalization in melanoma are the use of FA-SiO_2_@AuNPs in A375 [28] and a nanoliposomal system in B16-F10 [29].

DOX is a chemotherapeutic drug used to treat different cancers such as bladder, breast, bone marrow, and lung cancer, among others [30]. However, its clinical use is limited due to severe side-effects that lead to a great impact on the quality of life of the patient and fragilizes the immune system, making the patient more susceptible to infections and diseases [30]. Numerous studies have investigated nanocarriers capable of simultaneously enabling cell imaging and DOX delivery [31,32,33]. However, to our best knowledge, the use of UCNPs for chemotherapeutic drug delivery has been rarely explored, especially when functionalized with FA, and no studies have reported this combination applied to melanoma, underscoring the novelty of the present work. However, to our best knowledge, the use of UCNPs for chemotherapeutic drug delivery has been rarely explored, especially when functionalized with FA, and no studies have reported this combination applied to melanoma, underscoring the novelty of the present work. Here, it is reported the synthesis and characterization of UCNPs coated with a DOX-loaded mesoporous silica shell bearing folate ligands. A systematic study of the effects of UCNPs, DOX, and UCNPs loaded with DOX on the viability and cell cycle dynamics was carried out on A375, B16-F10, MNT-1, and SK-MEL-28 melanoma cell lines. This approach allowed us to assess both the cytotoxicity of UCNP@mSiO_2_-FA nanocarriers loaded with DOX and the cell cycle effects on melanoma cell lines.

## 2. Results and Discussion

### 2.1. Characterization of UCNP@mSiO_2_-FA

#### 2.1.1. Size of the Nanoparticles

**Figure 1:**
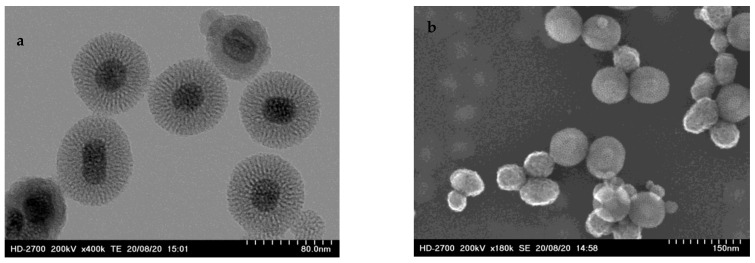
UCNP@mSiO_2_-FA physicochemical characterization. (**a**) TEM image; (**b**) SEM image.

**Figure A1:**
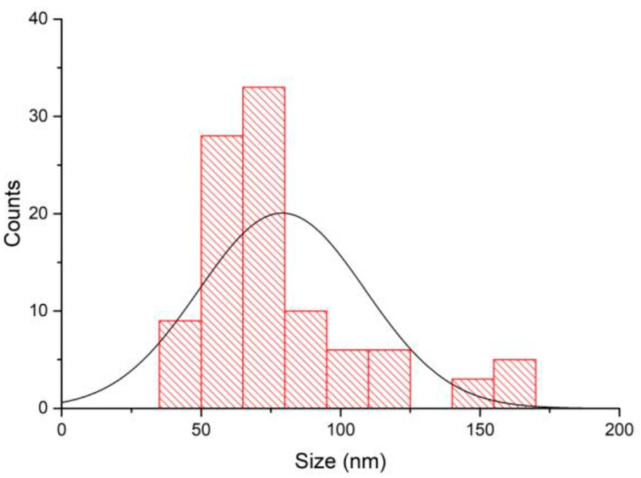
UCNP@mSiO_2_-FA particle size distribution (n = 100 particles).

UCNP@mSiO_2_-FA were characterized using transmission electron microscopy (TEM) and scanning electron microscopy (SEM). TEM images revealed an individual NaYF_4_:Yb,Er cores covered by a mesoporous silica coating, confirming the core–shell structure (Figure 1a). SEM analysis (Figure 1b) shows a spheric shape with an average size of 75 ± 29 nm (Figure A1).

#### 2.1.2. Surface Functionalization and Characterization

**Figure 2:**
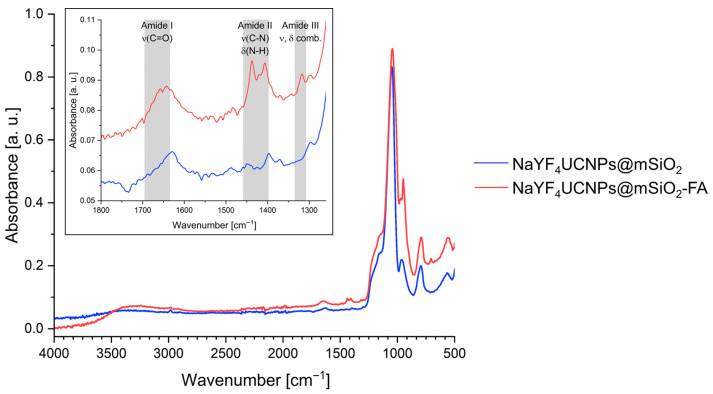
FTIR spectra of UCNP@mSiO_2_ (NaYF_4_:Yb/Er@mSiO_2_) and UCNP@mSiO_2_-FA (NaYF_4_:Yb/Er@mSiO_2_-FA). Inset figure as a close-up of the spectra (1250 to 1800 cm^−1^) demonstrating amide peaks.

**Figure A2:**
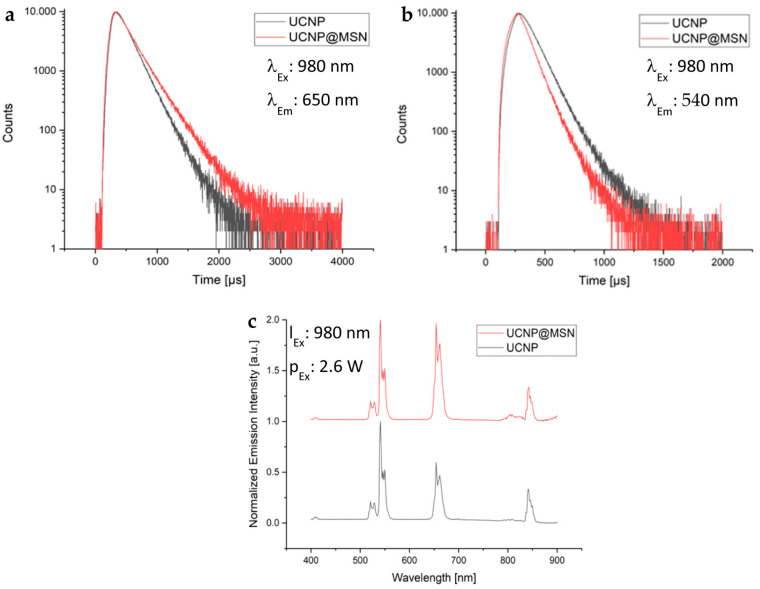
Optical characterization data of bare UCNP (black) and UCNP@MSN (red). (**a**) Upconversion luminescence lifetime spectra (*λ_Ex_*: 980 nm) at *λ_Em_*: 650 nm, (**b**) Upconversion luminescence lifetime spectra (*λ_Ex_*: 980 nm) at *λ_Em_*: 540 nm, and (**c**) Upconversion emission spectra (*λ_Ex_*: 980 nm; p_*Ex*_: 2 W).

The successful functionalization of UCNP@mSiO_2_ with FA was first assessed by Fourier-transform infrared spectroscopy (FTIR) spectroscopy. After functionalization with FA, the FTIR spectrum shows several new peaks at 1650, 1439–1407, and 1317 cm^−1^, as highlighted in Figure 2. These peaks can be assigned to the Amide I (C=O stretch), Amide II (C–N stretch and N–H bend), and Amide III (combination of stretching and bending) vibrations, respectively [34]. For further corroboration, elemental analysis is performed (Table 1). Compared to the unfunctionalized particles (UCNP@mSiO_2_), the UCNP@mSiO_2_-FA particles exhibited an increase in carbon (C) content, from 4.6 to 5.9%, hydrogen (H), from 2 to 3.3%, and nitrogen (N), from 0.07 to 0.69%. This increase is consistent with the presence of FA and supports its successful conjugation to the nanoparticle surface. Upconversion emission spectra as well as luminescence lifetime spectra for the emission lines at 540 nm and 650 nm of bare UCNP and UCNP@MSN were determined, as shown in Figure A2. The silica mesoporous coating modifies the luminescence behaviour of UCNPs. For the red emission at 650 nm (Figure A2a), UCNP@mSiO_2_ displays a longer lifetime and higher emission intensity, indicating reduced surface-related non-radiative quenching and improved radiative efficiency. In contrast, the green emission at 540 nm (Figure A2b) exhibits a shorter lifetime after coating, consistent with additional non-radiative pathways introduced by –OH vibrational modes in the silica shell. Overall, the coating selectively affects different Er^3+^ energy levels, enhancing red upconversion while modulating green emission dynamics, leading to a more efficient and stable luminescent response.

#### 2.1.3. Hydrodynamic Diameter and Surface Charge in Biological and Aqueous Media

Dynamic light scattering (DLS) and electrophoretic mobility measurements were performed to assess the hydrodynamic diameter (Dh), polydispersity index (PdI), and surface charge of UCNP@mSiO_2_-FA nanoparticles in different microenvironments, i.e., dispersed in water and in DMEM, at concentrations of 25 and 100 μg/mL (Table 2). The Z-average hydrodynamic diameter was slightly lower in DMEM than in deionized water (dH_2_0). Nevertheless, the primary size population (Dh peak 1) is considerably higher than for nanoparticles dispersed in H_2_0. Notably, the primary size population (Dh Peak 1) detected in water displayed smaller values (e.g., ~80 nm at 25 μg/mL) compared to DMEM, where larger aggregates were observed (Dh > 250 nm). These differences likely reflect the formation of protein corona and ionic interactions in the cell culture medium, which alter nanoparticle dispersion behaviour. Regarding the polydispersity index (PdI), only the samples dispersed in water exhibited values below 0.35, which is generally considered indicative of acceptable size homogeneity. In contrast, the higher PdI values observed in DMEM (> 0.67) suggest a broader size distribution and potential agglomeration in this medium.

Zeta potential values were consistently negative in both media, ranging from –10.47 to –15.47 mV, which falls below the conventional threshold for electrostatic colloidal stability (|ζ| > 30 mV). Therefore, these nanoparticles may rely more on the steric stabilization (e.g., surface ligands) than purely on surface charge to prevent aggregation, particularly under physiological conditions.

### 2.2. Loading of Doxorubicin

The DOX loading capacity of FA-functionalized nanoparticles was evaluated, yielding an average of drug loading of 91.92 ± 1.78 μg of DOX per mg of UCNP@mSiO_2_-FA (Table A1). This corresponds to a loading efficiency of 74.40 ± 1.21% (Table A1). These results are slightly higher than those reported in previous studies involving UCNP-based nanocarriers. However, it is important to note that such comparisons must consider differences in experimental protocols—particularly nanoparticle concentration—which can markedly influence loading performance. For instance, the loading efficiency of DOX in hollow mesoporous structured luminescent UCNPs was 59% (4 mg/mL nanoparticle concentration) [35], while UCNP-based and PEGylated organo–silica hybrid micelles reached 65% [36].

### 2.3. Doxorubicin Release

**Figure 3:**
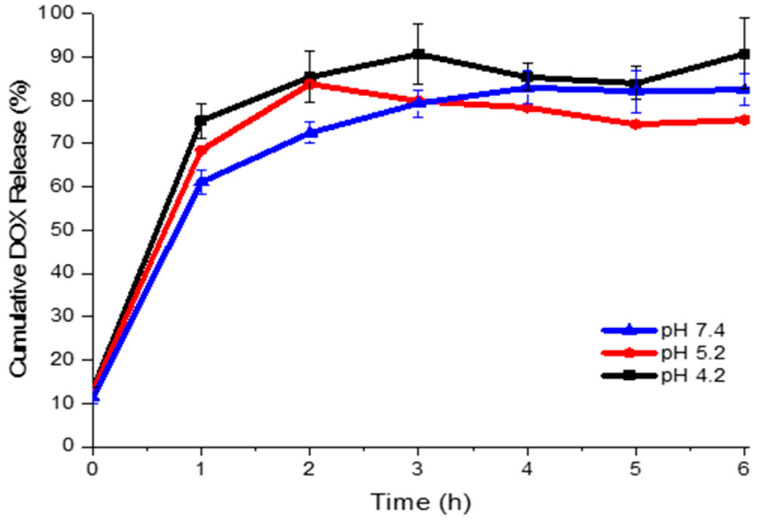
Cumulative release of DOX from UCNP@mSiO_2_-FA-DOX at pH 4.5, 5.2, and 7.4.

Figure 3 shows the cumulative release profile of DOX from UCNP@mSiO_2_-FA-DOX under three different pH conditions: 5.2 and 4.5 (representing the acidic tumoral environment) and 7.4 (physiological pH). A rapid and pronounced release was observed within the first hour, followed by a plateau after approximately 6 h, maintaining a steady release behaviour for all pH conditions. After 6 h, the cumulative DOX release reached 90.8% at pH 4.2, 75.5% at pH 5.2, and 82.5% at pH 7.4. These findings suggest a modest pH-responsive behaviour, with a slightly higher release in acidic conditions, which may be beneficial for preferential drug release in tumour tissues.

The initial rapid release is likely due to the desorption of weakly adsorbed DOX molecules on the external surface of the nanoparticles surface. This burst release could be advantageous for cancer cell therapy by delivering an initial cytotoxic dose capable of rapidly affecting cancer cells, as suggested in previous studies [37]. The subsequent slower release phase, sustained over time, may help to maintain effective intracellular drug concentrations, contributing to decreased cancer cell proliferation and reducing the probability of cancer recurrence [37], as observed in other DOX-loaded nanocarriers [33,38,39,40].

### 2.4. Cell Viability

**Figure 4:**
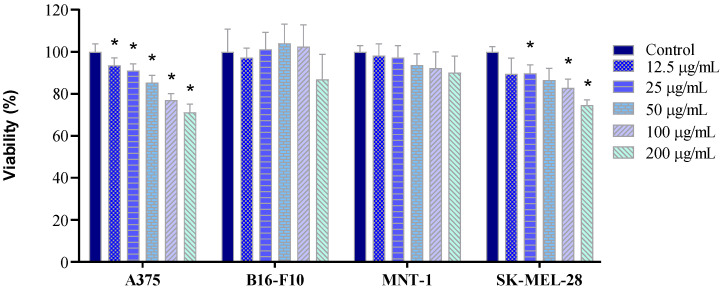
Cell viability (%) of A375, B16-F10, MNT-1, and SK-MEL-28 cells, measured by the WST-8 assay, after 24 h exposure to UCNP@mSiO_2_-FA (12.5, 25, 50, 100, and 200 μg/mL). Bars represent the standard error. Statistically significant differences (*p* < 0.05) in relation to the control group are represented as (*).

Subsequently, cell exposure studies were performed, using four cell lines: A375 (amelanotic human melanoma), B16-F10 (pigmented murine melanoma), MNT-1 (pigmented human melanoma), and SK-MEL-28 (lightly pigmented human melanoma). After exposure of the four cell lines to the UCNPs@mSiO_2_-FA, cell viability was determined. For cell lines A375 and SK-MEL-28, a significant dose-related cytotoxicity is observed (Figure 4—A375 and SK-MEL-28). Both A375 (Figure 4—A375) and SK-MEL-28 (Figure 4—SK-MEL-28) cell lines show statistically significant differences for every concentration, except for two conditions in SK-MEL-28, at 12.5 and 50 μg/mL. Both MNT-1 and B16-F10 cell lines did not demonstrate statistically significant differences (Figure 4—B16-F10 and MNT-1). These results demonstrate the biocompatibility of UCNP@mSiO_2_-FA for all cell lines, as cell viabilities remained above 70% at a concentration of 100 μg/mL, consistent with reports in the literature for other cell lines [41,42]. Some differences in sensitivity among the cell lines were observed, with the amelanotic A375 and SK-MEL-28 cell lines being slightly more sensitive than the highly pigmented B16-F10 and MNT-1 cells, as previously reported [43,44].

**Figure 5:**
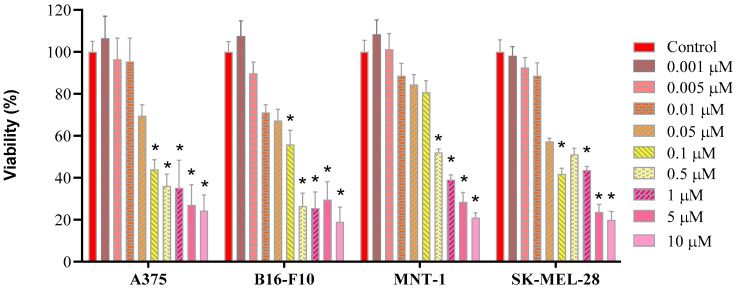
Cell viability (%) of A375, B16-F10, MNT-1, and SK-MEL-28 cells, measured by the WST-8 assay, after 24 h exposure to free DOX (0.001; 0.005; 0.01; 0.5; 1; 5; 10 µM). Bars represent the standard error. Statistically significant differences (*p* < 0.05) are represented as (*) in relation to the control group.

Next, the effect of free DOX on cell viability is determined (Figure 5). Free DOX shows a dose-dependent action on the percentage of viable cells of each cell line. Viability above 88% was observed in all cell lines when exposed to 0.01 μM of DOX, except for B16-F10, revealing a viability above 70%. For higher concentrations, a drastic decrease in cellular viability is observed, where viability is compromised (bellow 70%) from the concentration of 0.1 μM of DOX for A375, B16-F10, and SK-MEL-28 cell lines, and from 0.5 μM of DOX for MNT-1. This response to DOX has also been observed in other publications [45,46].

**Figure 6:**
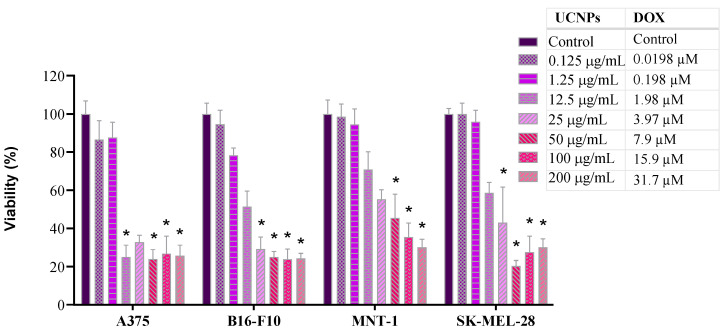
Cell viability (%) of A375, B16-F10, MNT-1, and SK-MEL-28 cells, measured by the WST-8 assay, after 24 h exposure to UCNP@mSiO_2_-FA-DOX (0.125, 1.25, 12.5, 25, 50, 100, and 200 μg/mL) and the correspondence of loaded DOX. Bars represent the standard error. Statistically significant differences (*p* < 0.05) in relation to the control group are represented as (*).

Subsequently, the cytotoxicity of the nanoparticles loaded with DOX was examined (Figure 6). As expected, the UCNP@mSiO_2_-FA-DOX exhibited a concentration-dependent cytotoxicity in all cell lines. A375 cell viability decreased significantly at UCNP@mSiO_2_-FA-DOX concentrations of 1.98 µM and above, whereas for B16F10 and SK-MEL-28 cells, a comparable reduction was observed only at concentrations exceeding 3.97 µM. In the case of MNT-1 cells, a significant decrease in viability occurred only at the highest concentrations tested (≥ 7.93 µM UCNP@mSiO_2_-FA-DOX).

From the results of the cell viability studies (Figure 5 and Figure 6), the 20% inhibitory concentration (IC_20_) and half maximal inhibitory concentration (IC_50_) were determined for free DOX and UCNP@mSiO_2_-FA-DOX (Table 3). The A375 and B16-F10 cell lines showed comparable IC_50_ values under both free DOX and UCNP@mSiO_2_-FA-DOX, while MNT-1 demonstrated the highest resistance to both free DOX and UCNP@mSiO_2_-FA-DOX, consistent with the results observed for UCNP@mSiO_2_-FA. This suggests that the melanin production by each cell line may be associated with the response to DOX exposure. Melanin is known to bind approximately 900 nmol/mg DOX, decreasing in vivo drug activity while conferring an increased resistance [47].

Across all tested cell lines, IC_50_ values for free DOX were consistently lower than those for DOX loaded into UCNP@mSiO_2_-FA. This observation is consistent with previous reports. Tomankova and co-workers [48] demonstrated markedly reduced cytotoxicity for a doxorubicin-loaded superparamagnetic iron oxide nanoassembly (IC_50_ = 18.245 μM) compared with free DOX (IC_50_ = 1.2009 μM). Likewise, Wang and co-workers [49] reported that UCNP–DOX exhibited a higher IC_50_ value (12 μM) than free DOX (3 μM). Similarly, Yang et al. [50], using FA-free SPIO/DOX-loaded polymer vesicles in HeLa cells, also observed greater cytotoxicity for free DOX than for the nanocarrier-bound formulation, further confirming the consistently greater cytotoxicity of free DOX. The higher toxicity of free DOX compared with UCNP@mSiO_2_-FA-DOX can be attributed to differences in their cellular uptake mechanisms. Free DOX enters cells primarily by passive diffusion and rapidly accumulates in the nucleus, where it intercalates DNA. In contrast, nanoparticle-bound DOX is predominantly internalized via endocytic pathways and released within acidic endo-lysosomal compartments, leading to delayed intracellular availability and reduced immediate cytotoxicity [48,49].

### 2.5. Cell Cycle

Since tumour cells are characterized by an unregulated cell cycle that results in uncontrolled cell proliferation, cell cycle disruption analysis is normally selected for cancer therapy utilizing therapeutic agents that target components of these pathways [51]. DOX binds to DNA-associated proteins, leading to a reduction in DNA replication and RNA transcription [52]. This also leads to an activation of the apoptosis pathway, resulting in a cell cycle arrest at G2/M stages [53]. Considering this, the analysis of the cell cycle dynamics seemed to be very pertinent.

**Figure 7:**
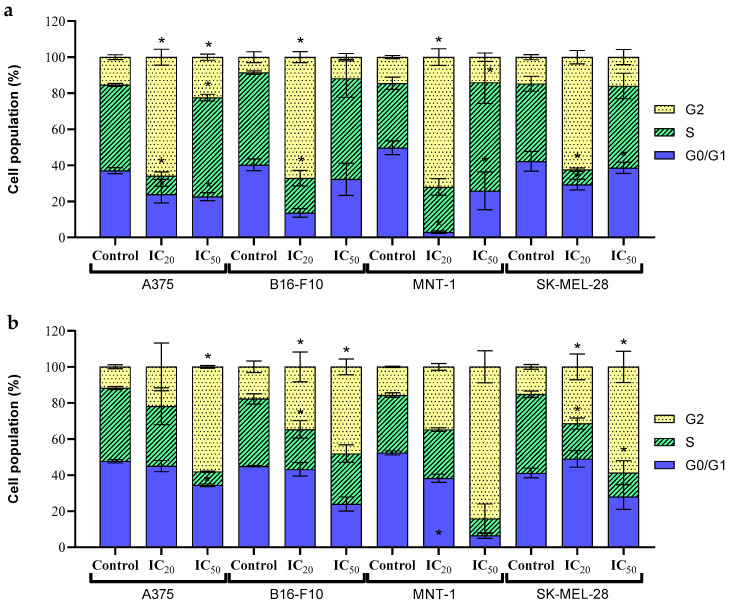
Cell cycle dynamics of A375, B16-F10, MNT-1, and SK-MEL-28 cells after 24 h exposure to (**a**) free DOX or (**b**) UCNP@mSiO_2_-FA-DOX at IC20 and IC50 (calculated in Table 3), measured by flow cytometry. Results are expressed as mean ± SD. Statistically significant differences (*p* < 0.05) in relation to the control group are represented as (*).

Analysis of the cell cycle profile of all cell lines when exposed to free DOX demonstrated some variations. Concerning the G0/G1 phase, both ICs (calculated from the viability results of free DOX and displayed in Table 3) had significantly less percentage of cells in this phase when compared to the control, in all cell lines, except for B16-F10, where no significant difference was observed. As for phase S, a significant decrease in the percentage of cells was observed in IC_20_ for all cell lines except for MNT-1, where it remained similar to the control. On the other hand, a significant increase in S phase was observed in the IC_50_ for cell lines A375 and MNT-1, but for B16-F10 and SK-MEL-28, there were no significant differences when compared to the control. When it comes to the G2 phase, there was a significant increase in the percentage of cells at this phase in A375 for both ICs, and in B16-F10 and MNT-1 for IC_20_, while no significant variation was observed for the other conditions and the remaining cell line, when compared to the negative control (Figure 7a). After an exposure of cells to DOX, the % of cells in G0/G1 and G2 phases has been reported to increase, while the % of cells in S phase has been observed to decrease [45]. This was observed after the exposure of the IC_20_ of free DOX to the different cell lines, as reported above. However, an increase in the S phase after exposure to the IC_50_ occurs. This may be due to some sort of protection mechanism of the cells to a higher concentration of DOX, to prevent further deleterious damage. Furthermore, DOX can bind directly to proteins of the cell membrane, triggering enzymatic electron reduction in DOX, leading to the formation of ROS free radicals [30].

Exposure to UCNP@mSiO_2_-FA-DOX resulted in a decrease in the percentage of cells at the G0/G1 phase for IC_50_ in all cell lines, with a statistically significant decrease for the A375 cell line. Concerning the S phase, both A375 and MNT-1 demonstrated no significant variance in the percentage of cells when compared to the control. As for B16-F10 and SK-MEL-28, both cell lines had similar results, where for the first line, only the IC_20_ induced a statistically significant decrease, while for SK-MEL-28, both ICs induced a statistically significant decrease in the percentage of cells in phase S, when compared to the control. As for the G2 phase, all cell lines demonstrated an increase in the percentage of cells when compared to the control, in both ICs (Figure 7b). The MNT-1 cell line showed no statistically significant difference between treatments, while for the A375 cells, only the IC_50_ exhibited a significant effect. For B16-F10 and SK-MEL-28, both ICs induced a significant increase compared to the control. These results are coherent to the findings reported for other nanoplatforms used in DOX delivery [54,55].

## 3. Materials and Methods

### 3.1. Chemicals

Dimethyl sulfoxide (DMSO; ≥99.7%), CCK-8 reagent, folic acid (97%), (3-Aminopropyl)triethoxysilane (APTES; 97%), N-Hydroxysuccinimide (NHS; 98%), Cetrimonium bromide (CTAB; 98%), tetraethoxysilane (TEOS; 98%), ammonia solution (25 wt% NH_3_ in water), Yttrium chloride hexahydrate (YCl_3_·6H_2_O; 99%), Ytterbium chloride hydrate (YbCl_3_·6H_2_O; 99%), Erbium chloride hexahydrate (ErCl_3_·6H_2_O; 99%), oleic acid (90%), Sodium hydroxide (NaOH; 98%), 1(-3-dimethylaminopropyl)-3-ethylcarbodiimide hydrochloride (EDC; 98%) and L-arginine (>98.5%) were purchased from Sigma-Aldrich (St. Louis, MO, USA). Dulbecco’s Modified Eagle’s Medium (DMEM), fetal bovine serum (FBS), L-glutamine, and fungizone (250 U/mL) were purchased from Gibco, Life Technologies (Grand Island, NY, USA). Penicillin–streptomycin (10,000 U/mL) was purchased from Grisp (Porto, Portugal). RNase and propidium iodide (PI, ≥94%) were purchased from Merck KGaA (Darmstadt, Germany). Ethanol PA (99.8%) was purchased from Fluka (Buchs, Switzerland), doxorubicin hydrochloride (≥98%) was purchased from Cayman Chemical (Ann Arbor, MI, USA), and ammonium nitrate (NH_4_NO_3_; 98%) was purchased from Chemlab (Zedelgem, Belgium). Methanol (HPLC grade), cyclohexane (p.a., ACS, ≥99.5%), chloroform (HPLC grade, ≥99.9%), and ethanol (99%) were purchased from Chemsolute (Renningen, Germany), and 1-octadecene (90%) and ammonium fluoride (NH_4_F; 90%) were purchased from Alfa Aesar (Waltham, MA, USA).

### 3.2. Synthesis of UCNP

The upconversion nanoparticles (UCNP) were prepared via a thermal decomposition method according to [56]. This synthesis was performed via in situ preparation of the nanoparticle precursors, followed by high-temperature growth of the nanoparticles. Briefly, YCl_3_·6H_2_O (1183.10 mg, 3.80 mmol), YbCl_3_·6H_2_O (387.50 mg, 1.00 mmol), and ErCl_3_·6H_2_O (38.17 mg, 0.20 mmol) were dissolved in 5 mL of methanol using sonication. This solution was then added to a mixture of oleic acid (40 mL) and 1-octadecene (80 mL) in a 250 mL three-necked flask. The reaction mixture was stirred and heated to 150 °C under a flow of argon. After 30 min, a vacuum was applied for an additional 30 min at the same temperature to remove residual low-boiling impurities. The mixture containing the rare-earth precursors was then cooled to room temperature under continuous argon flow. Subsequently, a methanolic solution (10 mL) containing NaOH (500 mg, 12.5 mmol) and NH_4_F (740 mg, 20 mmol) was added. The resulting suspension was heated to 120 °C for 30 min to evaporate excess methanol. The reaction mixture was then heated to 325 °C under reflux and a gentle argon flow, kept at this temperature for 30 min, and finally cooled to room temperature. The resulting UCNPs were purified according to a reported procedure [57], dispersed in cyclohexane, and stored at 4 °C.

### 3.3. Coating of UCNP with a Mesoporous Silica Shell (UCNP@mSiO_2_)

Coating of the UCNP with a mesoporous silica shell was performed according to the literature procedure [58]. Thereby, 0.4 g of cetyltrimethylammonium bromide (CTAB) was dissolved in 80 mL of water, then 2 mL of UCNP suspension in cyclohexane (20 mg/mL) was added to the aqueous CTAB solution, keeping the total UCNP concentration in water at 0.5 mg/mL. The mixture was stirred for 48 h at room temperature to evaporate cyclohexane and obtain the CTAB-stabilized UCNP. For the coating process, 20 mL of water, 3 mL of EtOH, and 24 μL of ammonia solution (25% *w*/*w*) were mixed in a 100 mL flask. Then, 10 mL of the CTAB-stabilized UCNPs (see above) were added to the mixture and heated to 70 °C under stirring at 600 rpm (Hettich Rotina 380R, Hettich, Kirchlengern, Germany). Subsequently, a mixture of 75 μL of TEOS in 4 mL of ethanol was added by the aid of a peristaltic pump (addition rate 0.5 mL/min). The reaction was kept at 70 °C for 1 h. The resulting UCNP@mSiO_2_ nanoparticles were washed three times with ethanol. For the washing step, ethanol was added to the dispersion to sediment the nanoparticles, and then the dispersion was centrifuged at 1108 g for 30 min. Finally, the surfactant (CTAB) was removed from the silica shell by an ion exchange process. The mesoporous silica-coated upconversion nanoparticles (UCNP@mSiO_2_) were added to a solution of 50 mL of ethanol and 0.3 g of NH_4_NO_3_ and kept at 60 °C for 2 h. The resulting UCNPs@mSiO_2_ were then washed by centrifugation and stored in ethanol.

Alternatively, the same particles were synthesized using a modified procedure from a different reference [59]. 1 mL of UCNP (59 mg/mL in cyclohexane) was diluted with 9 mL of chloroform, and the resulting mixture was sonicated with a probe sonicator (6 mm Probe diameter, Amplitude 70%). Then, it was added to a mixture of 500 mg of CTAB dissolved in 100 mL of Milli-Q water and sonicated for 15 min using a bath sonicator and 20 min using a probe sonicator (6 mm Probe diameter, Amplitude 70%), followed by vigorous stirring at room temperature for 20 h. Next, the organic solvents were evaporated from the turbid, white emulsion by heating it to 80 °C for 2 h under stirring. After another probe sonication step (6 mm Probe diameter, Amplitude 70%), 100 mL of Milli-Q water was added, and the resulting clear solution was heated at 85 °C for 20 min. Then, 65 mg of L-Arginine was added, followed by the dropwise addition of 800 µL of TEOS. After stirring for 3 h at 80 °C, the UCNP@mSiO_2_ nanoparticles were collected by centrifugation (25 min at 11,000 g), washed 2× with ethanol using centrifugation (25 min at 11,000 g) and redispersion steps. For extracting the organic template from the pores, the particles were redispersed in 50 mL of an ethanolic solution of NH_4_NO_3_ (1 g/50 mL), heated to reflux for 1 h, centrifuged (25 min at 11,000 g), and washed 1x with ethanol. This extraction step was repeated one more time. Afterwards, the particles were washed 2× with ethanol and stored in ethanol for further use.

### 3.4. Functionalization of UCNP@mSiO_2_ with Folic Acid

Functionalization with FA was performed following a literature method [60] with minor modifications. Briefly, 3 mg of FA, 15 µL of APTES, 3.3 mg of NHS, 4.8 mg of EDC, and 9 mL of DMSO were mixed and stirred for 1 h 30 min. Meanwhile, 31.5 mg of UCNP@mSiO_2_ nanoparticles were centrifuged at 10,400 g (Spectrafuge 24D, Labnet, Edison, NJ, USA) for 20 min, the supernatant was discarded, and the pellet was resuspended in 1 mL DMSO. The suspension was then sonicated in an ultrasound bath for 2 min. Next, 14.75 mL of DMSO was added, and the solution was sonicated using an ultrasonic probe for 10 min while kept on ice. Afterwards, 6.25 mL of the previously prepared FA solution was added, and the mixture was sonicated for an additional 2 h on ice. The nanoparticles were then collected by centrifugation (10 min at 16,300 g), the supernatant was discarded, and the pellet was resuspended in 0.5 mL of DMSO with sonication. This centrifugation step was repeated twice more, replacing DMSO with ethanol p.a. in the second cycle. Finally, the resulting solution was transferred to a watch glass and incubated overnight at 40 °C. In the end, UCNP@mSiO_2_-FA was obtained.

### 3.5. Nanoparticle Characterization

The morphology and size of UCNP@mSiO_2_-FA were analyzed using a 200 kV Hitachi HD-2700 STEM microscope (Hitachi, Tokyo, Japan) equipped with energy-dispersive X-ray spectroscopy (EDS) and secondary electron detectors. The average particle size was measured in ImageJ by calculating the area and diameter of 100 individual nanoparticles. To identify the composition of the UCNP@mSiO_2_-FA and evaluate the presence of amide groups after conjugation with FA, the samples were analyzed by Fourier Transform-Infrared Spectroscopy (FTIR). FTIR spectra were measured in the solid state. The spectra were collected using a Bruker optics tensor 27 spectrometer coupled to a horizontal ATR cell, using 256 scans at a resolution of 4 cm^–1^. Elemental analysis of carbon, nitrogen, and hydrogen performed to confirm the presence of FA on the UCNP@mSiO_2_-FA suspension was obtained on a Leco Truspec-Micro CHNS 630-200-200 (LECO, St. Joseph, MI, USA). The hydrodynamic diameter (Dh) and polydispersity index (PdI) of the nanoparticles were measured by dynamic light scattering (DLS), while the zeta potential was evaluated by electrophoretic mobility measurements. Both analyses were conducted using a ZetaSizer Nano ZS (Malvern Instruments, Malvern, UK) equipped with a 633 nm He-Ne laser. Dh was measured in Milli-Q water and DMEM, at concentrations of 25 μg/mL and 100 μg/mL, using three independent replicates for each condition. The determination of the zeta potential was performed using the same concentrations and dispersing media as the Dh.

### 3.6. Doxorubicin Loading

DOX hydrochloride solution (150 μg/mL) was prepared in ultra-pure water at pH 6. In parallel, 2.5 mg of UCNP@mSiO_2_-FA was weighed into each of 6 microtubes and dispersed in 1 mL of ultra-pure water (pH 6) under sonication. Then, 1 mL of the DOX solution was added to each nanoparticle suspension. The mixtures were vertically stirred (Heidolph Reax 2, 30 rpm) for 24 h, at room temperature in the dark. Following incubation, the nanoparticles were separated by centrifugation (4500 g, 5 min, Spectrafuge 24D, Labnet), and the residual DOX concentration ([DOX]_final_) was determined via UV-Vis spectrophotometry (480 nm, Cintra 303, GBC). The loading efficiency and the nanoparticle capacity were calculated using Equations 1 and 2, respectively. The final DOX-loaded nanocomposite (UCNP@mSiO_2_-FA-DOX) was recovered from the resulting pellet.

(1) $$\mathrm{Loading}\;\mathrm{Efficiency}\;\left(\%\right)=\frac{{\left[\mathrm{DOX}\right]}_{\mathrm{initial}\;}-{\left[\mathrm{DOX}\right]}_{\mathrm{final}}}{{\left[\mathrm{DOX}\right]}_{\mathrm{initial}}}\;\times \;100$$

(2) $$\mathrm{Nanoparticle}\;\mathrm{Capacity}\;(m_{\mathrm{DOX}}/m_{\mathrm{NP}})=\frac{m_{\mathrm{loaded}\;\mathrm{doxorubicin}}}{m_{\mathrm{nanoparticles}}}$$

### 3.7. Drug Release Profile

To evaluate the DOX release profile, UCNP@mSiO_2_-FA-DOX was suspended in 10 mL of sodium phosphate buffer (0.2 M), at pH 7.4, 5.2, or 4.2. The suspensions were vertically stirred (Heidolph Reax 2, 30 rpm) for 48 h at 37 °C. At predetermined time intervals, hourly for the first 6 h, then at 24 h, 30 h, and 48 h, 1 mL of the solution was withdrawn and replaced by an equal volume of fresh buffer. The nanoparticles were separated by centrifugation, and the supernatant was collected and analyzed using UV-VIS spectrophotometry (480 nm, Cintra 303, GBC) to assess DOX concentration.

### 3.8. Cell Culture

The melanoma cell lines A375, B16-F10, and SK-MEL-28 were purchased from the European Collection of Authenticated Cell Cultures (ECACC 88113005), and the pigmented human melanoma cell line MNT-1 was kindly provided by Dr. Manuela Gaspar (iMed.ULisboa, Portugal). Cells were maintained in cell culture flasks, in Dulbecco’s modified Eagle’s medium (DMEM), supplemented with 10% (*v*/*v*) Fetal Bovine Serum (FBS), 2 mM L-glutamine, 1% penicillin-streptomycin (10,000 U/mL), and 1% fungizone (250 U/mL), in a humidified atmosphere, at 37 °C with 5% CO_2_. Cell lines were subcultured when 80% confluency was reached.

### 3.9. Cell Viability Evaluation

Cell viability was determined by the WST-8 Cell Proliferation Kit. The concentration of cells seeded in each well varied within the used cell line: A375—35,000 cells/mL, B16-F10—25,000 cells/mL, MNT-1—35,000 cells/mL, and SK-MEL-28—50,000 cells/mL. After seeding, the plates were incubated for 24 h at 37 °C with 5% CO_2_. Then, the cell culture medium was replaced by either: (a) fresh medium as a control, (b) fresh culture medium with UCNP@mSiO_2_-FA at different concentrations (12.5; 25; 50; 100 and 200 μg/mL), (c) free DOX (0.001; 0.005; 0.01; 0.05; 0,1; 0.5; 1; 5; and 10 μM), or (d) UCNP@mSiO_2_-FA-DOX (0.125; 1.25; 12.5; 25; 50; 100 and 200 μg/mL). Cells were again incubated for 24 h. Afterwards, the medium of each well was replaced with 100 μL of fresh culture medium plus 10 μL of CCK-8 reagent. Plates were then incubated for 2 h, at 37 °C with 5% CO_2_. The absorbance value was measured at 450 nm with a microplate reader (Synergy HT Multi-Mode, BioTek, Winooski, VT, USA). Cell viability was calculated through Equation (3).

(3) $$\mathrm{Cell}\;\mathrm{viability}=\left(\frac{(\mathrm{Sample}\;\mathrm{Abs}\;-\;\mathrm{Blank}\;\mathrm{Abs})}{(\mathrm{Control}\;\mathrm{Abs}\;-\;\mathrm{Blank}\;\mathrm{Abs})}\right)\;\times \;100$$

### 3.10. Cell Cycle Analysis

Concerning the cell cycle analysis, the four cell lines were seeded in 12-well plates and left to attach for 24 h. Then, the cell culture medium was replaced with fresh medium with UCNP@mSiO_2_-FA-DOX and free DOX at the respective IC_20_ and IC_50_ values determined for each cell line. The cells were then incubated at 37 °C for 24 h and thereafter trypsinized, collected, centrifuged at 1157 g for 6 min, resuspended with 85% ethanol, and stored at - 20 °C until further analysis. For the cell cycle analysis, cells were centrifuged (3000 g for 6 min, Eppendorf 5804R) and the pellets were resuspended in 800 μL of PBS. Then, for each sample, 50 μg/mL RNase and 50 μg/mL propidium iodide (PI) were added, and the samples were incubated for at least 20 min, in the dark and at room temperature. The relative light scatter properties, side and forward scatter, as well as the relative fluorescence intensity of propidium iodide-stained nuclei, were measured with an Attune^®^ Acoustic Focusing Cytometer (Applied Biosystems, Thermo Fischer Scientific, Waltham, MA, USA) flow cytometer. Four replicates were performed for each treatment, and for each sample, a minimum of 5000 events were acquired. The percentage of cells in G0/G1, S, and G2 phases was determined using the FlowJo software v10 (Tree Star Inc., Ashland, OR, USA).

### 3.11. Statistical Analyses

For the WST-8 assays, two to three independent assays with four replicates each were performed. For the cell cycle analysis, one independent assay was performed with three to four replicates. All data are expressed as mean ± standard deviation (SD). Statistica 7.0 software was used for statistical analyses. All data were first tested for normality and homogeneity of variance, and transformed, if necessary, to meet statistical demands. For parametric results, one-way Analysis of Variance (ANOVA), followed by Dunnett’s test as Post hoc comparison, was applied to comparisons between control and treated groups. For non-parametric results, the Kruskal–Wallis test was applied to comparisons as stated before. In all analyses, differences between means were considered significant for *p* < 0.05 [61].

## 4. Conclusions

In conclusion, UCNP@mSiO_2_-FA-DOX nanocomposites were successfully designed for targeted DOX delivery to melanoma cancer cells and demonstrated efficient DOX loading. While UCNP@mSiO_2_-FA showed high biocompatibility across four different melanoma cell lines, UCNP@mSiO_2_-FA-DOX exhibited an enhanced cytotoxicity compared to UCNP@mSiO_2_-FA, with a clear dose-dependent response. The four cell lines used in this study displayed varying sensitivities to free DOX and to UCNP@mSiO_2_-FA-DOX. Cell cycle analysis revealed that the proportion of cells arrested in the G2/M phase increased with higher concentrations of UCNP@mSiO_2_-FA-DOX. Overall, this study increased the knowledge about the possibility of applying these nanoformulations as efficient drug delivery systems to melanoma therapy. Possible future studies could include further testing of these nanocomposites in mice to evaluate the nanoparticles’ efficiency in reducing or eliminating cancer cells.

## Figures and Tables

**Table 1:** Elemental analysis of UCNP@mSiO_2_ and UCNPs@mSiO_2_-FA.

Sample	% C	% H	% N
UCNP@mSiO_2_	4.646	1.998	0.069
UCNP@mSiO_2_-FA	5.865	3.251	0.688

**Table 2:** Z-average results, PdI, and zeta potential of UCNP@mSiO_2_-FA dispersed in dH_2_0 and DMEM at concentrations of 25 and 100 μg/mL. The results are shown as average ± standard deviation.

	DMEM		dH_2_O	
	25 μg/mL	100 μg/mL	25 μg/mL	100 μg/mL
**Dh (nm)**	82.1 ± 24.91	114.77 ± 13.71	174 ± 20.31	123.73 ± 13.07
**Peak 1 Area (%)**	66.07 ± 2.45	77.03 ± 3.02	96.77 ± 1.01	99.63 ± 0.64
**Dh Peak 1 (nm)**	305.37 ± 11.11	257.93 ± 19.45	79.57 ± 5.48	124.86 ± 13.71
**PdI**	0.676 ± 0.044	0.826 ± 0.068	0.342 ± 0.10	0.300 ± 0.011
**Zeta (mV)**	−15.47 ± 0.74	−13.07 ± 1.63	−10.47 ± 0.61	−10.8 ± 0.56

**Table 3:** Determined IC_20_ and IC_50_ values from the dose–response curves, for A375, B16-F10, MNT-1, and SK-MEL-28 cell lines, after 24 h exposure to free DOX (µM) and to UCNP@mSiO_2_-FA-DOX (μg/mL) and the correspondence of loaded DOX (µM).

		Free DOX	UCNP@mSiO_2_-FA-DOX	
			(µg/mL)	(µM DOX)
**A375**	**IC_20_** **IC_50_**	0.018	0.567	0.091
		0.168	7.111	1.138
**B16-F10**	**IC_20_** **IC_50_**	0.010	0.924	0.148
		0.120	9.794	1.567
**MNT-1**	**IC_20_** **IC_50_**	0.070	6.220	0.995
		0.575	41.701	6.672
**SK-MEL 28**	**IC_20_** **IC_50_**	0.010	2.598	0.416
		0.200	13.187	2.110

## Data Availability

The original contributions presented in this study are included in the article. Further inquiries can be directed to the corresponding author.

## Appendix A

The particle size distribution of UCNP@mSiO_2_-FA was determined from SEM images, as shown in Figure A1.

The DOX loading capacity of FA-functionalized nanoparticles was evaluated, as shown in Table A1.

**Table A1:** Functionalized UCNP@mSiO_2_-FA loading efficiency of DOX.

Initial [DOX] (μg/mL)	% Efficiency	Loaded DOX (μg/mg)
145.57	74.83	92.25
	73.92	94.32
	75.85	92.72
	73.97	91.58
	72.40	91.59
	75.18	88.91

Upconversion emission spectra as well as luminescence lifetime spectra for the emission lines at 540 nm and 650 nm of bare UCNP and UCNP@MSN were determined, as shown in Figure A2.
